# Needle Stick Injury Among Healthcare Workers in a Tertiary Care Setting in Dehradun, Sub-Himalayan Region: A Four-Year Record-Based Study

**DOI:** 10.7759/cureus.58448

**Published:** 2024-04-17

**Authors:** Rajender Singh, Garima Mittal, Abhay Srivastava

**Affiliations:** 1 Department of Microbiology, Himalayan Institute of Medical Sciences, Swami Rama Himalayan University, Dehradun, IND; 2 Department of Community Medicine, Himalayan Institute of Medical Sciences, Dehradun, IND

**Keywords:** hepatitis b, occupational health hazard, health-care worker, hiv diseases, needle stick and sharp injury

## Abstract

Introduction: Needlestick injuries (NSIs) represent a significant occupational health risk in healthcare settings. These injuries, caused by contaminated sharps such as needles, vials, and scalpel blades, can lead to percutaneous exposure to infectious materials. Despite the severity of NSIs, they often go unreported, highlighting a critical gap in occupational safety protocols.

Aims: This study aimed to investigate the occurrence of NSIs among healthcare workers (HCWs) by sex, profession, and working areas. It also sought to explore the underlying reasons for these injuries and the factors contributing to their underreporting.

Methodology: Adhering to the RECORD guidelines (Reporting of studies Conducted using Observational Routinely Collected Data), this record-based study involved a retrospective analysis of reported NSIs. Data were collected from voluntary reports by HCWs who experienced NSIs or exposure to potentially infectious materials such as blood and body fluids. Statistical analysis was conducted using IBM SPSS Statistics for Windows, Version 16 (Released 2007; IBM Corp., Armonk, New York) and Microsoft Excel 2010 (Microsoft Corporation, Redmond, Washington).

Results: Data from 142 participants indicated a higher proportion of females experiencing NSIs compared to males, with rates of 57.7% pre-COVID and 60.6% during COVID. There were notable shifts in NSI rates across professions, with increases observed among staff nurses and ward attendants/helpers. Analysis of injury circumstances revealed a decrease in sampling procedure-related injuries but an increase during intravenous procedures and biomedical waste segregation. Injuries occurring on the right-hand index finger decreased from 52.1% pre-COVID to 31% during COVID, while those on the left-hand index finger increased from 19.7% pre-COVID to 39.4% during COVID. Statistically significant associations were found between the injury site and the place of occurrence (p=0.021). Healthcare professionals commonly cleansed the site with disinfectants and used personal protective equipment (PPE) kits, with increased PPE usage noted during the COVID-19 pandemic. These findings emphasize the evolving dynamics of NSIs among HCWs and underscore the importance of tailored preventive measures during pandemics.

## Introduction

Needlestick injuries (NSIs) represent a significant occupational health hazard in healthcare settings, resulting from contact with needles or sharp objects such as hypodermic needles, blood collection needles, intravenous stylets, and needles used in intravenous delivery systems. These injuries, characterized by breaks in skin continuity caused by contaminated needle sticks or infected sharps like vials and scalpel blades, pose a risk of percutaneous exposure for healthcare workers (HCWs).

HCWs face an elevated risk of NSIs due to their work environment, with potential exposure to blood-borne pathogens like hepatitis B virus (HBV), hepatitis C virus (HCV), and HIV, particularly when handling patients' blood, urine, and saliva [1]. The United States Centers for Disease Control and Prevention estimates an annual occurrence of 600,000 to 1,000,000 NSIs, with infection risks accounting for 0.2-0.5% for HIV, 3-10% for HCV, and 40% for HBV [2,3].

Major cases of NSIs often result from improper use of personal protective equipment (PPE) such as gloves, attempts to recap needles, improper sharps disposal, and the insertion of IV (intravenous) cannulas and drips. Inadequate work experience and low knowledge of blood-borne infections also contribute to NSI occurrences among HCWs.

NSIs vary among HCWs based on workplace and environmental factors. Studies suggest that approximately 40% of NSIs in the past three months and 75% in the past year have gone unreported, posing a significant barrier to exposed HCWs receiving post-exposure prophylaxis and leading to serious consequences [4].

Literature review

Numerous studies have investigated reasons for the underreporting of NSIs among HCWs, citing factors such as unawareness of reporting systems, heavy workloads, fear of repercussions including job loss, and inadequate understanding of the importance and hazards associated with NSIs [5-7]. Further studies are needed to assess HCWs' behavior regarding NSI reporting.

The recent COVID-19 pandemic has not only impacted global lifestyles but also brought significant changes to the healthcare system. The pandemic highlighted HCWs' vulnerability to occupational hazards and emphasized the importance of ensuring their safety [8].

The use of PPE during the pandemic not only protected against infectious agents but also contributed to a reduction in NSI incidence [9]. The pandemic heightened awareness of health, hygiene, and preventive measures, emphasizing the importance of precautions against occupational risks to effectively fulfill healthcare duties.

This study aims to report NSI incidence in our hospital over the past four years (2018-2021) and compare results between the pre-pandemic and pandemic eras. It also examines the circumstances, locations, and sites of NSIs, incorporating routine anti-HBsAg titer testing conducted for NSI individuals. The study uniquely analyzes NSI occurrence and consequences across two distinct timeframes.

## Materials and methods

Study design

This study conforms to the guidelines set forth by the Reporting of Studies Conducted Using Observational Routinely Collected Health Data (RECORD), ensuring rigorous reporting standards for healthcare studies utilizing routinely collected data [10]. It is a record-based investigation conducted in alignment with RECORD guidelines, scrutinizing reported occurrences of NSIs within a tertiary care hospital in Dehradun. The study period encompasses the onset of the COVID-19 pandemic in late 2019, distinguishing NSIs reported on or before December 31, 2019, as pre-COVID, and those reported on or after January 1, 2020, as during COVID-19 times to assess the impact of the COVID-19 pandemic on infection control practices.

Inclusion criteria****

HCWs from various departments including doctors, nurses, lab technicians, ward attendants, and housekeeping staff were eligible for inclusion. Participation in the study required HCWs to provide informed consent for the use of their data.

Exclusion criteria****

Those who did not provide informed consent were excluded from the study.

Variables

Blood samples collected from HCWs underwent screening for HIV-1 and HIV-2, testing for hepatitis B surface antigen (HBsAg), antibodies against the hepatitis C virus, and hepatitis B surface antibodies titer (anti-HBs titer). These tests were performed as part of the baseline tests for those who reported NSIs because it may later be difficult to attribute whether the infection was acquired due to this occupational exposure or any prior exposure. The study's sample size is contingent upon the number of self-reported NSIs in the hospital during the specified four-year period, resulting in a sample size of 142.

Data sources and analysis

Data retrieval involved extracting information from past HICC-NSI records spanning from 2018 to 2021. Before the commencement of the study, informed consent was secured from reporting HCWs, alongside ethical clearance from the institute’s ethics committee. Screening for HIV-1, HIV-2, HBsAg, and antibodies against HCV adhered to national guidelines. Statistical analysis was performed using IBM SPSS Statistics for Windows, Version 16 (Released 2007; IBM Corp., Armonk, New York) and Microsoft Excel 2010 (Microsoft Corporation, Redmond, Washington), with a significance level set at P<0.05.

Bias

Given its record-based nature, bias is mitigated; however, potential bias may manifest during HCWs' completion of questionnaires due to apprehension of consequences. Fear of job loss or other repercussions could influence reporting accuracy, while non-reporting events may introduce bias.

Quantitative variables

Data categorization was based on HCWs' sex, profession, working area, circumstances, and site of injury. Further stratification occurred based on patient status (known/unknown) and HCWs' anti-HBs titer presented the data in number and percentage.

## Results

Descriptive data

The demographic characteristics of the 142 study participants who experienced NSIs and reported the events to HICC are presented in Table 1. A higher proportion of females (57.7% in the pre-COVID period and 60.6% during the COVID period) reported NSIs compared to males (42.3% in the pre-COVID period and 39.4% during the COVID period).

This study sheds light on various aspects related to NSIs occurring in the hospital, offering a comparison between the pre-COVID and during COVID periods. It highlights how the profession of HCWs correlates with NSI occurrence and provides insight into frequently injured areas and body sites.

The profession-wise distribution indicates a decrease in exposed doctors (from 21.1% to 11.3%) and an increase in affected staff nurses (from 25.4% to 31%). Nursing students experienced NSIs both pre-COVID (14.1%) and during COVID times (8.5%). Lab/OT technicians showed no significant change. Resident doctors and ward attendants/helpers demonstrated fluctuations in NSI occurrences (Table 1).

**Table 1:** Baseline characteristics of study participants SR/JR: senior resident/junior resident, OT: operation theater, ICU: intensive care unit

Category	Details	Pre-COVID, N (%)	During COVID, N (%)	Chi-square/P-value
Gender wise distribution	Male	30 (42.3)	28 (39.4)	0.0291/0.86
	Female	41 (57.7)	43 (60.6)	
Profession wise distribution	Doctors	15 (21.1)	8 (11.3)	8.416/0.209
	Staff nurse	18 (25.4)	22 (31)	
	Nursing students	10 (14.1)	6 (8.5)	
	Lab/OT technicians	7 (9.9)	7 (9.9)	
	Residents (SR/JR)	12 (16.9)	15 (21.1)	
	Ward attendants/helpers	4 (5.6)	11 (15.5)	
	Housekeeping staff	5 (7)	2 (2.8)	
Place of occurrence	Emergency	4 (5.6)	4 (5.6)	5.843/0.441
	OT	17 (23.9)	9 (12.7)	
	ICU	5 (7)	11 (15.5)	
	Lab and Blood Bank	7 (9.9)	6 (8.5)	
	Surgical Department	7 (9.9)	6 (8.5)	
	Medicine Department	23 (32.4)	29 (40.8)	
	Others	8 (11.3)	6 (8.5)	
Circumstances of injury	During sampling procedure	18 (25.4)	6 (8.5)	10.43/0.03
	During intravenous administration (recapping)	16 (22.5)	25 (35.2)	
	During surgical procedure	31 (43.7)	2 (2.8)	
	Biomedical waste segregation	6 (8.5)	14 (19.7)	
	Others	0 (0)	24 (33.8)	
Site of injury reported	Right-hand index finger	37 (52.1)	22 (31)	12.69/0.013
	Right thumb	4 (5.6)	7 (9.9)	
	Left-hand index finger	14 (19.7)	28 (39.4)	
	Left thumb	5 (7)	9 (12.7)	
	Others	11 (15.5)	5 (7)	

In terms of injury occurrence locations, NSIs in the emergency department remained constant, while the ICU witnessed an increase. NSIs in the operating theater (OT), labs, blood bank, surgical department, and medicine department showed variable trends between the pre-COVID and during COVID periods. Other hospital areas also displayed fluctuations in NSI occurrences (Table 1).

Analyzing the circumstances of injury revealed changes in patterns. Sampling procedure-related injuries decreased during COVID-19 times, while injuries during intravenous procedures increased. NSIs during surgical procedures drastically declined during the COVID phase. Biomedical waste segregation-related NSIs showed an increase, along with various other circumstances causing injuries during COVID-19 times (Table 1).

Data analysis based on the site of injury indicated changes in patterns. Injuries on the right-hand index finger decreased, while those on the left-hand index finger increased. Injuries on the thumbs also showed varying trends between the pre-COVID and during COVID periods, along with a decrease in injuries on other body parts like the left and right palms of the hand (Table 1).

Statistical significance

A statistically significant association was found between the site of injury and the place where the injury occurred (p=0.021) (Table 2).

**Table 2:** Association between the site of injury and the place where the injury occurred OT: operation theatre, ICU: intensive care unit

Place	Site of injury, N (%)					Chi-square/P-value
Ward	Right-hand index finger	Right Thumb	Left-hand index finger	Left thumb	Other	
Emergency	3 (5.1%)	0 (0%)	1 (2.4%)	4 (28.6%)	0 (0%)	40.073/0.021
OT	17 (28.8%)	1 (9.1%)	2 (4.8%)	4 (14.3%)	4 (25%)	
ICU	5 (8.5%)	1 (9.1%)	8 (19.0%)	1 (7.1%)	1 (6.3%)	
Ward lab and blood bank	3 (5.1%)	1 (9.1%)	7 (16.7%)	1 (7.1%)	1 (6.3%)	
Surgical department	8 (13.6%)	0 (0%)	3 (7.1%)	0 (0%)	2 (12.5%)	
Medicine department	19(32.2%)	7 (63.6.%)	16(38.1%)	5 (35.7%)	5 (31.3%)	
Others	4 (6.8%)	1 (9.1%)	5 (11.9%)	1 (7.1%)	3 (18.8%)	

Table 3 compares NSI data based on the site of injury across both COVID-19 phases. The majority of cases involved the index finger of the right hand, with a significant difference noted in NSIs affecting both hand index fingers (Table 3).

**Table 3:** Association between the site of injury and the time period of COVID

Site of injury	Period		Chi-square/P-value
	Pre-COVID	During COVID	
Right-hand index finger	37(62.7%)	22(37.3%)	6.524/0.011
Right thumb	4(36.4%)	7(63.6%)	0.887/0.532
Left-hand index finger	14(33.3%)	28(66.7%)	6.627/0.010
Left thumb	5(35.7%)	9(64.3%)	1.268/0.399
Others	11(68.8%)	5(31.2%)	2.536/0.183

Table 4 presents data on hepatitis B vaccination status and anti-HBs titer of exposed HCWs, revealing variations between pre-COVID and during COVID times (Table 4).

**Table 4:** Hepatitis B vaccination status and anti-HBs titer of NSI-exposed HCWs Anti-HBs: hepatitis B surface antibody, NSI: needlestick injury, HCW: healthcare worker

Era	Year	Unvaccinated/incomplete vaccination	Complete vaccination (all three doses)	Anti-HBs titer		
				>10 mIU/mL	<10 mIU/mL	Not done (ND)
Pre-COVID	2018	6	20	10	2	8
	2019	10	46	20	3	22
During COVID	2020	12	28	14	3	11
	2021	10	21	12	4	5
Total		38	104	56	12	46

The study also examined whether the source status of the injury to the exposed worker was known or not. Cases with unknown source status decreased during COVID-19 times, while known and reactive cases exhibited a decline. Known and non-reactive cases increased during COVID-19 times (Table 5).

**Table 5:** Source status (known/unknown) of viral markers among NSI-exposed HCWs NSI: needlestick injury, HCW: healthcare worker, HIV: human immunodeficiency virus, HBV: hepatitis B virus, HCV: hepatitis C virus

Era	Year	Unknown	Known and reactive			Known and non-reactive
			HIV	HBV	HCV	
Pre-COVID	2018	1	1	3	0	21
	2019	5	3	3	7	27
During COVID	2020	0	1	2	2	35
	2021	3	0	2	5	21
Total		9	5	10	14	104

**Figure 1:**
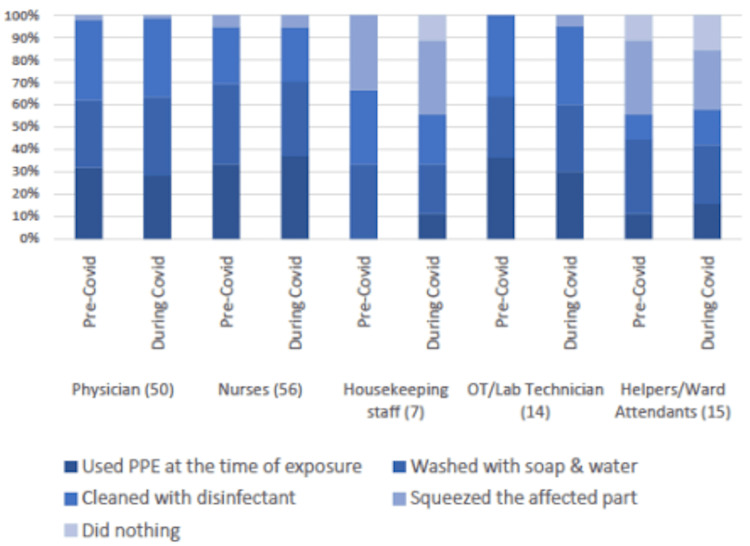
Healthcare professional's behavior: pre-COVID and during COVID PPE: personal protective equipment

Healthcare professionals' behavior post-exposure was documented, with most cleansing the site with disinfectant or soap and water and utilizing PPE kits during exposure. The usage of PPE kits increased during the COVID times, as illustrated in Figure 1.

**Figure 2:**
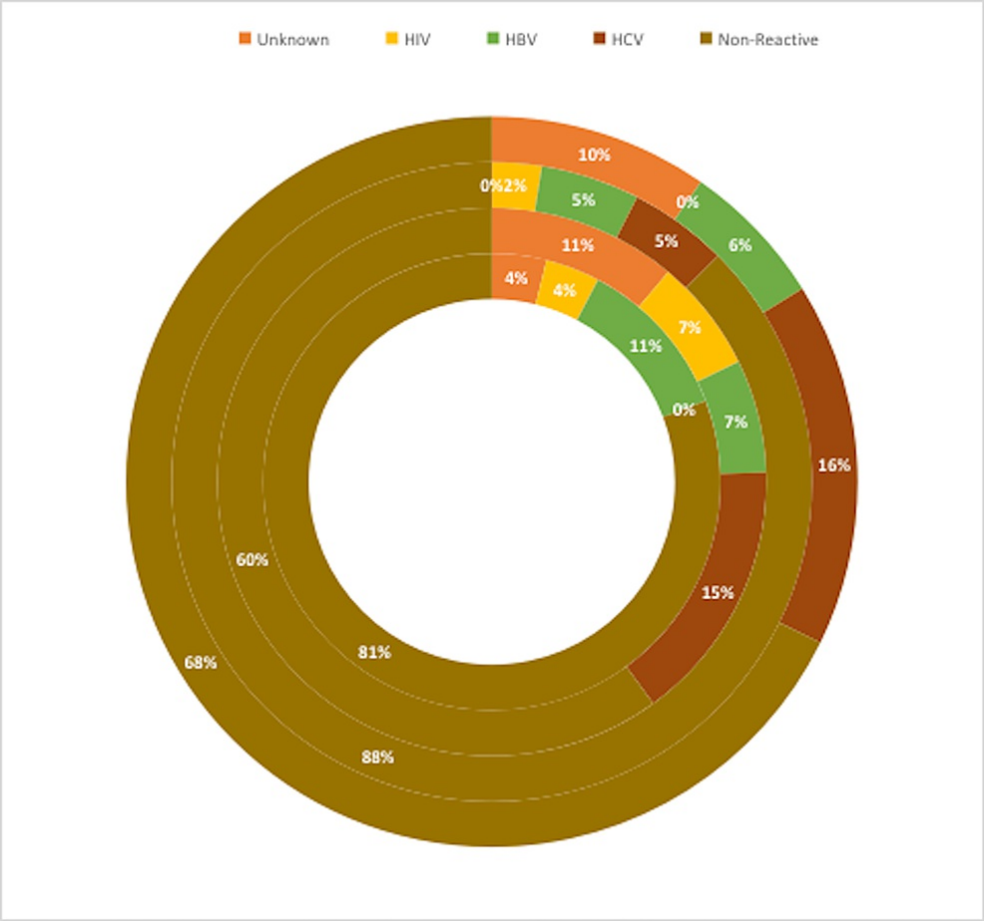
Reactive/non-reactive status of the source Outer to inner pie representing the years 2021, 2020, 2019, and 2018 HIV: human immunodeficiency virus, HBV: hepatitis B virus, HCV: hepatitis C virus

Figure 2 depicts the reactive and non-reactive status of the source patient (Figure 2).

## Discussion

Occupational hazards are prevalent across various fields, prompting the implementation of preventive measures and regulations to mitigate accidents. NSIs pose a significant occupational risk, particularly for HCWs, leading to guidelines from organizations like the World Health Organization (WHO), the National Institute for Occupational Safety and Health (NIOSH), and the Occupational Safety and Health Administration (OSHA). NSIs serve as a route for the transmission of serious blood-borne infections such as HIV, Hepatitis B, and C, underscoring the importance of prevention to reduce transmission among HCWs [11].

The study provides a comparative analysis of NSI exposure in a tertiary care hospital before and during the COVID era. While no significant differences in exposure numbers were noted between the two periods, a slight decrease in NSIs among doctors was observed. This decline may be attributed to the proper utilization of PPE during the COVID period. Effective PPE usage not only prevented COVID-19 transmission but also reduced occupational accidents. Comprehensive provision of PPE kits and necessary protective measures to the entire healthcare staff during COVID-19 facilitated the delivery of optimal patient care. Additionally, the decrease in elective surgical interventions during COVID-19 contributed to the reduced number of exposures among doctors.

Female HCWs (59.2%) seem to be exposed more frequently to NSIs than males (41.2%) possibly due to the unequal gender distribution in this study, as nurses were mostly female; however, a few other studies also supported this finding [12,13].

The body parts most commonly involved in NSIs were the fingers, accounting for 56 cases (45.2%), others included the right and left thumb along with the palm of the hand. This finding aligns with research conducted in Saudi Arabia [14]. The prevalence of finger injuries could be attributed to their frequent involvement in tasks such as needle handling, recapping, suturing, and inserting intravenous lines.

Notably, the percentage of NSIs was higher among nurses compared to doctors, potentially due to their frequent close contact with patients and involvement in procedures utilizing sharp items, such as phlebotomy and intravenous needle insertion [15].

The exposure to sharps decreased among doctors while it increased among nurses, helpers, and ward attendants. The rise in injuries within these groups suggests potential lapses in proper PPE usage and a lack of carefulness during minor surgical procedures. The surge in patient numbers during the COVID period added to the workload of healthcare staff, many of whom were also infected, leading to increased instances of exposure, particularly among nurses. The heightened workload and decreased efficiency due to extended work hours were contributing factors to the increased incidence of NSIs.

Moreover, healthcare professionals across various fields are mandated to wear PPE due to the highly transmissible nature of COVID-19, while facing the challenge of caring for a surge of patients amidst extremely stressful conditions. Excessive use of PPE can impair vision and mobility, and when combined with the strain of increased workloads, the repetitive nature of medical procedures heightens the risk of NSIs and other potential health complications among HCWs [16].

This trend was also observed among resident doctors, possibly due to similar reasons. Instances of injuries during activities such as recapping and biomedical waste segregation notably increased during the COVID period, aligning with the overall rise in injuries among nurses, helpers, and ward attendants [17].

Moreover, various NSIs were reported without specific scenarios, possibly attributed to the increased layering of PPE. While increased PPE coverage offers protection, it can also reduce efficiency, leading to mishandling and injuries [18]. The decrease in exposure incidents from the operating theater during COVID-19 was due to fewer elective surgeries [19].

The study indicates that occupational hazards are more prevalent in developing countries due to factors such as insufficient staffing, long working hours, lack of experience, and inadequate educational programs [20-23]. Nurses consistently reported the highest number of incidents, highlighting the need for targeted training programs to prevent NSIs among them [24-26].

The aggressive vaccination approach has been effective in reducing the risk of hepatitis B virus (HBV) infection post-exposure [27,28]. While vaccines for HIV and HCV are unavailable, hepatitis B vaccination is essential. Regular monitoring of anti-HBs titers, indicating proper immune response post-vaccination, is crucial for HCWs [29]. Making hepatitis B vaccination mandatory for HCWs could significantly reduce the risk of infection [30].

However, the study has limitations, as it only considers reported incidences, overlooking many unreported cases due to negligence. Awareness programs should emphasize the importance of reporting NSIs and the potential consequences of not doing so, with workshops organized to address this issue.

## Conclusions

It can be inferred that the occurrence of NSIs is influenced by various factors rather than being solely dependent on one aspect. While the proper utilization of PPE during the COVID era reduced exposure rates in certain contexts, increased workload contributed to higher rates in other scenarios.

Furthermore, it is crucial to ensure HCWs are fully aware of the potential consequences of NSIs. Regular workshops should be conducted to educate them about associated risks, provide training on prevention strategies, emphasize adherence to guidelines, and encourage prompt reporting of incidents. HCWs should also be educated on the importance of correct PPE usage to mitigate the risk of NSIs effectively.
